# Axonal protection achieved by blockade of sodium/calcium exchange in a new model of ischemia *in vivo*

**DOI:** 10.1016/j.neuropharm.2012.04.019

**Published:** 2012-09

**Authors:** Fengfeng Bei, Kenneth J. Smith

**Affiliations:** Department of Neuroinflammation, The Institute of Neurology (Queen Square), University College London, 1 Wakefield Street, London WC1N 1PJ, United Kingdom

**Keywords:** Ischemia, Stroke, White matter injury, Axonal protection, Sodium/calcium exchanger, ET-1, endothelin-1, NCX, sodium/calcium exchanger

## Abstract

Ischemic white matter injury has been relatively little studied despite its importance to the outcome of stroke. To aid such research a new rat model has been developed *in vivo* and used to assess whether blockade of the sodium/calcium exchanger is effective in protecting central axons from ischemic injury. Vasoconstrictive agent endothelin-1 was injected into the rat spinal cord to induce ischemia. KB-R7943 or SEA0400 was administered systemically to block the operation of the sodium/calcium exchanger. Endothelin-1 caused profound reduction of local blood perfusion and resulted in a prompt loss of axonal conduction. Whereas recovery of conduction following vehicle administration was only to 10.5 ± 9% of baseline (*n* = 8) 4.5 h after endothelin-1 injection, recovery following KB-R7943 (30 mg/kg, i.a.) administration was increased to 35 ± 9% of baseline (*n* = 6; *P* < 0.001). SEA0400 (30 mg/kg, i.a.) was also protective (33.2 ± 6% of baseline, *n* = 4; *P* < 0.001). Neither drug improved conduction by diminishing the severity of the ischemia. The protective effect of KB-R7943 persisted for at least 3 days after ischemia, as it improved axonal conduction (76.3 ± 11% for KB-R7943 vs. 51.0 ± 19% for vehicle; *P* < 0.01) and reduced lesion area (55.6 ± 15% for KB-R7943 vs. 77.9 ± 9% for vehicle; *P* < 0.01) at this time. In conclusion, a new model of white matter ischemia has been introduced suitable for both structural and functional studies *in vivo*. Blocking the sodium/calcium exchanger protects central axons from ischemic injury *in vivo*.

## Highlights

► A new *in vivo* model in the rat for studying ischemic injury of the white matter. ► The model permits direct, serial functional examination of the axons. ► Protection of axons against ischemia by blocking the sodium/calcium exchanger.

## 1 Introduction

Ischemic stroke is a major cause of permanent, severe neurological deficits (Stroke Therapy Academic Industry Roundtable, 1999; Lo et al., 2003b). Although therapies such as thrombolysis can reduce the magnitude of the damage to CNS tissue by reducing the severity of the insult (The National Institute of Neurological Disorders and Stroke rt-PA Stroke Study Group, 1995), therapies that can protect the tissue from the consequences of the insult are very limited (See review Ginsberg, 2008). One concept for neuroprotective therapy is that after ischemia the CNS tissue exhibits a “penumbra” (Astrup et al., 1977), i.e. an area at the periphery of the infarct which is recruited over time, but which is potentially salvageable with timely intervention (Memezawa et al., 1992; Lovblad et al., 1997; Back et al., 2004). Although it is clear that most cases of stroke involve the white matter (Ho et al., 2005), and that the damage to the white matter can be at least as important in causing neurological deficits as damage to the gray matter (See reviews Dewar et al., 1999; Gladstone et al., 2002; Goldberg and Ransom, 2003; Pantoni, 2006; Bakiri et al., 2009), most preclinical studies have targeted the gray matter. This discrepancy is partly because the current rodent stroke models mainly involve the gray matter as the gray matter accounts for approximately 90% of the rodent brain in comparison with only 40–50% of the human brain (Zhang and Sejnowski, 2000). However, research into white matter protection has been hampered by a relative lack of experimental models, especially models *in vivo* that allow for functional examination of the axons. Here we introduce such a model in the rat spinal cord, in which axonal function can be monitored *in vivo* serially over hours, or over days. The ischemia results from the intraspinal injection of the vasoconstrictive agent endothelin-1 (ET-1) (Yanagisawa et al., 1988). ET-1 is an important physiological mediator, regulating vasomotor tone and blood pressure via its effects on the blood vessels. Produced and secreted by the endothelial cells, ET-1 acts on the vascular smooth muscle cells and triggers Ca^2+^-mediated smooth muscle contraction via the ET_A_ receptors (For review see Masaki et al., 1999). Exogenous ET-1 has been successfully applied to induce local cerebral ischemia in both the gray matter (Fuxe et al., 1992; Sharkey et al., 1993; Macrae et al., 1993) and white matter (Hughes et al., 2003; Frost et al., 2006; Sozmen et al., 2009).

One strategy for axonal protection formulated by Stys, Waxman, Ransom and colleagues (Stys et al., 1992b, 1993; Waxman et al., 1994), is based on the fact that axons deprived of oxygen accumulate Na^+^ within the axoplasm due to the electrochemical inward gradient for Na^+^ and the failure of adequate extrusion by the Na^+^/K^+^ ATPase (“sodium pump”) because of insufficient ATP. The raised intra-axonal Na^+^ concentration is believed to result in the detrimental influx of Ca^2+^ via reverse operation of the axolemmal sodium/calcium exchanger (NCX) (see below). In support of this scheme, axonal protection has been achieved by inhibiting Na^+^ entry in a number of models believed to involve energy insufficiency, including spinal cord injury (Agrawal and Fehlings, 1996; Hains et al., 2004) and models of multiple sclerosis (Kapoor et al., 2003; Lo et al., 2003a; Bechtold et al., 2004).

The NCX normally serves to export axoplasmic Ca^2+^ at the expense of importing Na^+^, powered by the electrochemical driving force for Na^+^ entry. However, if the intracellular concentration of Na^+^ rises, the axon becomes depolarized and the electrochemical Na^+^ gradient is therefore diminished, so that eventually the electrochemical driving force for Ca^2+^ entry becomes dominant and the NCX is driven in reverse mode, importing Ca^2+^ into the axoplasm. In the absence of sufficient energy for Ca^2+^ extrusion or sequestering, the Ca^2+^ accumulates and activates degradative pathways leading to axonal degeneration (For review see Annunziato et al., 2004). The axonal location of the NCX has been confirmed in both rat optic nerve and the dorsal columns by immunolabeling (Steffensen et al., 1997). Accordingly, inhibitors of the reverse-mode operation of the NCX are effective in protecting axons from anoxia or simulated ischemia *in vitro* (Stys et al., 1992b; Li et al., 2000), but whether this strategy is effective in axonal protection following true ischemia *in vivo* is not known. Here we have developed and employed a new model of ischemia to test the efficacy in axonal protection *in vivo* of two selective inhibitors of reverse-mode operation of the NCX, KB-R7943 (2-[2-[4-(4-nitrobenzyloxy)phenyl]ethyl]isothiourea methane-sulfonate) and SEA0400 (2-[4-[(2,5-difluorophenyl)methoxy]phenoxy]-5-ethoxyaniline).

## 2 Experimental procedures

Two protocols have been developed, one comprising an acute, terminal examination, allowing for more controlled studies on model characterization and initial “screening” of protective compounds, and the other a recovery preparation, which is clinically more relevant and can be used to determine whether axonal protection is durable. All the animal procedures conformed to the U.K. Animals (Scientific Procedures) Act, 1986 and associated guidelines.

### 2.1 Acute preparation

Adult rats (Sprague-Dawley; male; 280–350 g) were anesthetized (1.5–2% isoflurane in room air) and prepared for prolonged electrophysiological monitoring. Preparation included maintenance of rectal temperature (36.5 ± 0.5 °C) using a homeothermic blanket (Harvard Apparatus, UK) and continuous monitoring of both end-tidal carbon dioxide and breathing rate, and arterial blood pressure via cannulation of the right internal carotid artery, to ensure the adequacy of anesthesia. Artificial ventilation was introduced after the trachea was intubated and a neuromuscular blocking agent (gallamine triethiode; Concord Pharmaceuticals Ltd, Essex, UK; 40 mg/ml) administered as a bolus via the right internal carotid artery. Electrical stimulation (50 μs duration; DS2 stimulator, Digitimer Ltd, Hertfordshire, UK) was applied to the spinal cord via a platinum wire stimulating cathode (prepared in house). The cathode was implanted over the junction of the intact T8/T9 vertebrae, and the anode (a stainless steel needle electrode) was placed subcutaneously at the right shoulder. The evoked compound action potentials (CAPs) conducted antidromically via the dorsal columns were differentially recorded (Neurolog System, Digitimer Ltd, Hertfordshire, UK) every 2 min (averaged, *n* = 64) from two stainless steel needle electrodes positioned at the base (‘active’) and tip (‘indifferent’) of the tail. The stimulating voltage was gradually increased from zero until a maximal CAP was evoked, and then the applied voltage was selected as 1.5 times this value (typically 60–70 V). The recording period (six-hours) started with stimulation at 1 Hz for 60 min, followed by stimulation at 50 Hz for 210 min, returning to 1 Hz for the final 90 min. The stimulation at 50 Hz, which is within the physiological range for many axons (Prochazka and Gorassini, 1998), was designed to increase the metabolic demand of the axons. The magnitude of the CAP was normalized and expressed as a percentage of the CAP recorded at baseline (i.e. the average amplitude of the CAPs recorded over the first 30 min of stimulation, declared as 100%). Functional recovery of the axons was expressed as the normalized magnitude of the CAP recorded at *t* = 270 min. The CAPs obtained at each specified time point were averaged among animals.

To induce ischemia in the dorsal columns, ET-1 (2.28 nmol; Alexis, San Diego, CA, USA) was micro-injected bilaterally into the spinal cord (injections made ∼550 μm lateral to the midline, at depths of 200 μm, 600 μm, 1000 μm and 1400 μm from the surface; 8 × 1 μl in total) at the junction between the T11/T12 vertebrae, 90 min after the onset of the CAP recording (defined as T_0_). The injection was performed using a pre-pulled glass pipette (30–50 μm tip diameter) containing the liquid to be injected, connected by a segment of flexible cannula to a 20 ml air-filled disposable syringe (used to apply positive pressure to the pipette). The set of injections at each site took about 90 s, after which a 30-s interval was allowed before the glass injection pipette was fully withdrawn to prevent back-flow of the injected solution. The second set of injections on the contralateral side was initiated immediately to ensure bilateral inhibition of the blood flow in the spinal cord. The total injection process took about 4 min. Such injections of saline (controls) did not diminish the magnitude of the CAP continuously monitored along the dorsal columns. Because the dorsal columns in rat mainly receive their blood supply via the vascular plexus in the adjacent gray matter, probably via the post-capillary bed (Tveten, 1976), and the arterial vessels that respond to the constrictive effect of ET-1 are located in the gray matter (Tveten, 1976), the ET-1 injection here is intended to target the gray matter adjacent to the dorsal columns. Vascular perfusion of the cord at the injection site was continuously monitored using laser Doppler flowmetry (PeriFlux 3; Perimed, Sweden), and the severity of ischemia was assessed as the maximal reduction of perfusion expressed as a percentage of baseline. Because the dorsal vein located on the dorsal columns obscures the access of the Doppler probe for directly measuring the blood flow in the dorsal columns, the blood flow at one side of the adjacent gray matter was monitored to assess the effect of ET-1. The tip of the Doppler probe was gently placed on the surface of the cord and left in place throughout the recording (i.e. no re-positioning for injection). At the end of the electrical recordings the animals were either killed by anesthetic over-dose, or perfused transcardially with glutaraldehyde (4% in 0.15 M phosphate buffer, pH 7.4) for later histological processing.

KB-R7943 (30 mg/kg, *n* = 6), SEA0400 (30 mg/kg, *n* = 4), or vehicle (40% dimethyl sulfoxide or DMSO in saline, *n* = 8) were slowly administered intra-arterially (i.a.) over 10 min via the cannulated right internal carotid artery, 60 min prior to the induction of ischemia. KB-R7943 and SEA0400 were generous gifts from Eisai Ltd, UK.

### 2.2 Recovery preparation

Rats (*n* = 16) were anesthetized as described above and the rectal temperature maintained at 36.5 ± 0.5 °C. The trachea was intubated to allow monitoring of end-tidal carbon dioxide, and artificial ventilation, if required, following ET-1 injection. Axonal conduction along the dorsal columns was assessed by recording the CAP before any drug treatment (and prior to ET-1 injection), using a similar protocol to that described above. ET-1 (2.28 nmol) was injected bilaterally into the gray matter at the T13 vertebra using the protocol described above (onset of injection: T_0_). This first set of ET-1 injections was followed 60 min (T_60 min_) later by a second set of ET-1 injections (1.14 nmol) delivered ∼1 mm more caudally (∼550 μm lateral to midline, 1 μl injection at 200 μm and 600 μm depths; 4 × 1 μl in total). The injection protocol was demonstrated not to affect the function of the dorsal column axons by testing it using the acute preparation described above. The animals were allowed to recover from the anesthesia once spontaneous ventilation was reliable, and checked twice a day for post-operative care including manually emptying the bladder if urinary retention occurred.

In order to determine the time course of the recovery of blood flow after ET-1 injection, laser Doppler flowmetry was used repeatedly to measure the blood flow at the spinal injection site to obtain the “absolute” values at T_0_, T_10 min_, T_60 min_, T_Day1_, and T_Day3_, and all the values were normalized to the value at T_0_ for each animal. Apart from keeping all the parameters of the Doppler flowmeter consistent for each measurement, the following measures were also taken to minimize variability introduced by re-positioning the Doppler probe: first, the animal was anesthetized, and the vertebrae were fixed to isolate the spinal cord from ventilatory movements and the arrangement was held stable for at least half an hour before measurement; second, the probe was re-positioned at precisely the same site of the cord, guided by charcoal labeling and the pattern of pial blood vessels; third, large blood vessels on the cord surface were avoided at the measurement site; and fourth, for each measurement the maximal reading was noted after fine adjustments of the position of the probe on the cord. We found that although re-positioning of the Doppler probe will inevitably introduce variability of reading, the precautions above provided an acceptable repeatability in naïve animals, when performed by the same investigator. We therefore used this method to estimate the recovery of blood flow over 3 days.

For terminal examination, the rats were re-anesthetized (as above) at day 3 post-treatment and conduction along the dorsal column axons was assessed as before. The area under the CAP was expressed as a percentage of that obtained prior to ET-1 injection. The rats were then perfused transcardially with glutaraldehyde (4% in 0.15 M phosphate buffer, pH 7.4).

For drug treatment, KB-R7943 (30 mg/kg, *n* = 8) or vehicle (40% DMSO in saline, *n* = 8) were slowly administered intravenously (i.v.), 60 min before the injection of ET-1. To maintain blinding, the drugs were coded by an independent investigator and the animals were randomly assigned into each group. The electrophysiological examinations were performed blind to the drug treatment administered. The drug treatment was only revealed after all the electrophysiological and histological data had been obtained and analyzed.

### 2.3 Histology

A 2 cm length of the spinal cord containing the pre-labeled injection site was cut transversely into blocks of 0.5 mm, which were washed with 0.15 M phosphate buffer (15 min × 3), and treated with 1.5% buffered osmium tetroxide for 120 min. After washing (10 min × 3), the tissue blocks were dehydrated in a series of alcohols with ascending concentrations (15 min in 30% alcohol, 15 min in 50% alcohol, 15 min in 90% alcohol, 3 × 20 min in 100% alcohol). The tissues were placed in 100% propylene oxide for 30 min, followed by one repeat, then in 50% propylene oxide with 50% resin for 60 min, and in 25% propylene oxide with 75% resin for another 60 min, and finally in 100% resin overnight at 4 °C. The resin was polymerized in an oven (60 °C) for 24–36 h. The embedded spinal cord blocks were cut transversely into 1 μm sections and stained with toluidine blue (1%). Sections obtained from each tissue block were first examined grossly to identify the block exhibiting the most severe dorsal column lesion for each spinal cord. Here sections were selected for imaging and further analysis. Analysis of the transverse, ‘two-dimensional’ lesion size, instead of ‘three-dimensional’ lesion volume, was employed, based on the assumption that only the transverse extent of the lesion affects the number of conducting axons as all the axons run longitudinally along the dorsal columns. In the recovery study, such selected sections were used to quantify the area of the dorsal column lesion expressed as a percentage of the whole dorsal column area. The histological assessments were conducted blind. Two samples in the KB-R7943 recovery trial were excluded from histological analysis for technical reasons.

### 2.4 Statistics

Data were expressed as mean ± SD. Student's *t* test was used to calculate the statistical significance for single comparisons. ANOVA was used for multiple comparisons, followed by Bonferroni *post hoc* test. Spearman's rank correlation test was used for correlation analysis. *P* < 0.05 was considered statistically significant.

## 3 Results

### 3.1 Model of white matter ischemia: acute preparation

**Fig. 1:**
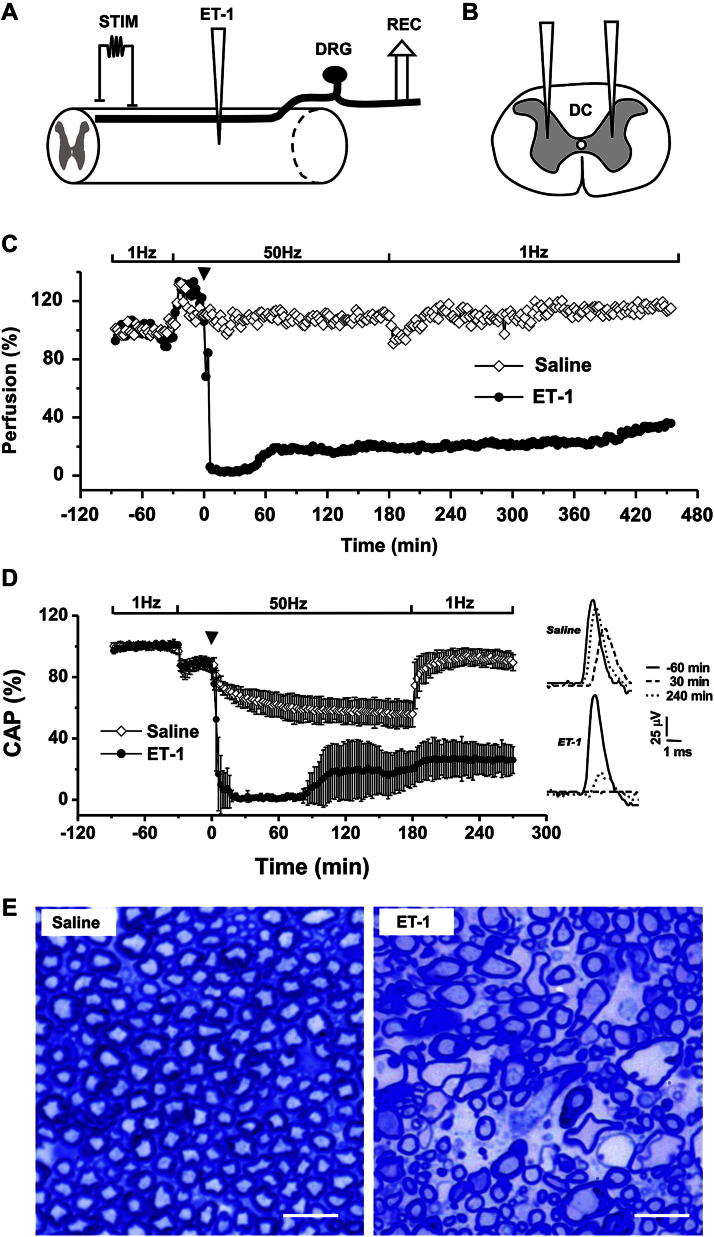
Data showing the acute model of ET-1-induced ischemia. Axonal conduction along primary afferent axons in the dorsal columns was continuously monitored (A). ET-1 injection was performed bilaterally at multiple injection sites (A & B) to induce ischemia within the dorsal columns. Blood perfusion of the spinal cord measured at the site of ET-1 injection, relative to baseline, is presented before and after the injection (time zero; C). Increase in the frequency of electrical stimulation from 1 Hz to 50 Hz resulted in an increase in blood flow, but this was profoundly reduced by the injection of ET-1 (2.28 nmol; arrowhead). Comparable saline injection had no similar effect. The injection of ET-1 caused a complete loss of the compound action potential (CAP) within 20 min (D), and the total blockade of conduction lasted for approximately 60 min. The amplitude eventually recovered to 18.5 ± 9% of baseline (*n* = 5) at *t* = 270 min. In the saline control group the amplitude of the CAP recovered to 93.9 ± 3% of baseline (*n* = 6) after the low frequency stimulation was resumed. Representative images of axons in the ventral portion of the dorsal columns are shown in transverse sections in (E) 4.5 h after saline or ET-1 injection, in tissue fixed at the end of electrical recording. The saline (control) axons appear normal throughout the dorsal columns, but the axons in the ventral portion of the dorsa columns are undergoing degeneration following ET-1 injection. Data in D: mean ± SD. STIM: stimulation. DRG: dorsal root ganglia. REC: recording. DC: dorsal columns. Bars in *E* = 10 μm.

The electrophysiological studies assessed the primary afferent axons in the dorsal column, as only the action potentials evoked in these fibers were recorded in the periphery (Fig. 1A). The magnitude of the evoked CAP along those fibers indicates the number of conducting axons (Cummins et al., 1979). Pilot experiments determined the optimal injection location (Fig. 1A & B) and the dose-dependence of ET-1 (0–2.28 nmol) on the persisting terminal electrophysiological deficit. Electrically active axons, which presumably have higher metabolic demand, were found to be more vulnerable to the ischemia, as animals that had experienced continuous stimulation at 50 Hz had significantly reduced recovery of the CAP compared with those that had experienced stimulation at 1 Hz throughout the experiment (Supplementary Fig. 1). Therefore, a period of 50 Hz stimulation (210 min) was applied to the axons in anesthetized rats in combination of ET-1 injection (2.28 nmol) in acute experiments.

Vascular perfusion of the spinal cord remained stable during stimulation at 1 Hz (Fig. 1C), but it increased promptly when the stimulation frequency was increased to 50 Hz, before falling more gradually toward a new steady level. The intraspinal injection of ET-1 resulted in a profound reduction of perfusion to below 10% of baseline within ∼10 min, and it remained at this level for ∼60 min before gradually increasing to achieve ∼20% of baseline 270 min following ET-1 injection (when most acute experiments end). Recovery of ∼40% of baseline was achieved 450 min following ET-1 injection (the longest time point recorded in some acute, terminal experiments). In contrast, the control intraspinal injection of saline had little effect on vascular perfusion (Fig. 1C).

Recordings of the CAP were stable during the period of stimulation at 1 Hz (Fig. 1D). When the stimulation frequency was increased to 50 Hz, the CAP diminished in amplitude by ∼10% to reach a new plateau. In controls, saline injection, along with continuous 50 Hz stimulation, further diminished the amplitude of the CAP until the stimulation frequency was returned to 1 Hz at *t* = 180 min. The final recovery of the amplitude of the CAP was 93.9 ± 3% of baseline (*n* = 6). Upon intraspinal injection of ET-1 the CAP promptly reduced in amplitude such that it was completely abolished within ∼10 min. Axonal conduction started to recover ∼90 min later after a partial recovery in perfusion which usually occurred approximately 60 min after ET-1 injection (Fig. 1C), suggesting that local perfusion has to recover to a certain level (∼20% of baseline) before axonal conduction recovers. By the end of the recording period, the amplitude of the CAP reached a stable level of 18.5 ± 9% of baseline (*n* = 5) after ET-1 injection. This final CAP recovery was significantly smaller than the recovery after saline injection (*P* < 0.001). The amplitude of the CAP at the end of the recording period (*t* = 270 min) was not significantly different from the amplitude at *t* = 210 min (paired Student's *t* test) in either saline or ET-1 group, suggesting that both recoveries had reached a plateau by the end of the experiment. Histological examination of the dorsal columns after ET-1 treatment (Fig. 1E) revealed significant pathology, especially of the myelinated axons in the ventral portion of the dorsal columns. Compared with the normal appearance of tightly-packed axons with intact myelin sheaths found throughout the dorsal columns in the saline-treated animals, many myelinated axons in animals injected with ET-1 appeared swollen, in an oedematous region containing distended myelin sheaths and expansion of the periaxonal space. The dorsal portion of the dorsal columns was relatively intact after ET-1 injection.

The intraspinal injection of ET-1 reduced the mean systemic arterial blood pressure by 0–50% (23 ± 24%), but this always returned to baseline within 150 min of ET-1 injection (Supplementary Fig. 2). To ensure that the effects on axonal conduction were not due to a direct ‘toxic’ effect of ET-1 on axons, ET-1 was injected directly into the dorsal columns rather than into the gray matter. No evidence of direct toxicity was detected (Supplementary Fig. 3).

### 3.2 Model of white matter ischemia: recovery preparation

The acute, terminal preparation allowed for continuous electrophysiological monitoring of the axons as the ischemia developed and evolved, but it was only feasible to study the ischemic axons for a few hours following ischemia. To permit examination after a longer period a recovery experiment was developed that allowed the evolution of the ischemic lesion over days, or longer periods. Since the local blood perfusion was estimated to recover to its full extent by 1 day after ET-1 injection (Supplementary Fig. 4), the animals were examined three days after ET-1 injection, in the belief that the acute phase of any damage would have become fully manifest at this time.

**Fig. 2:**
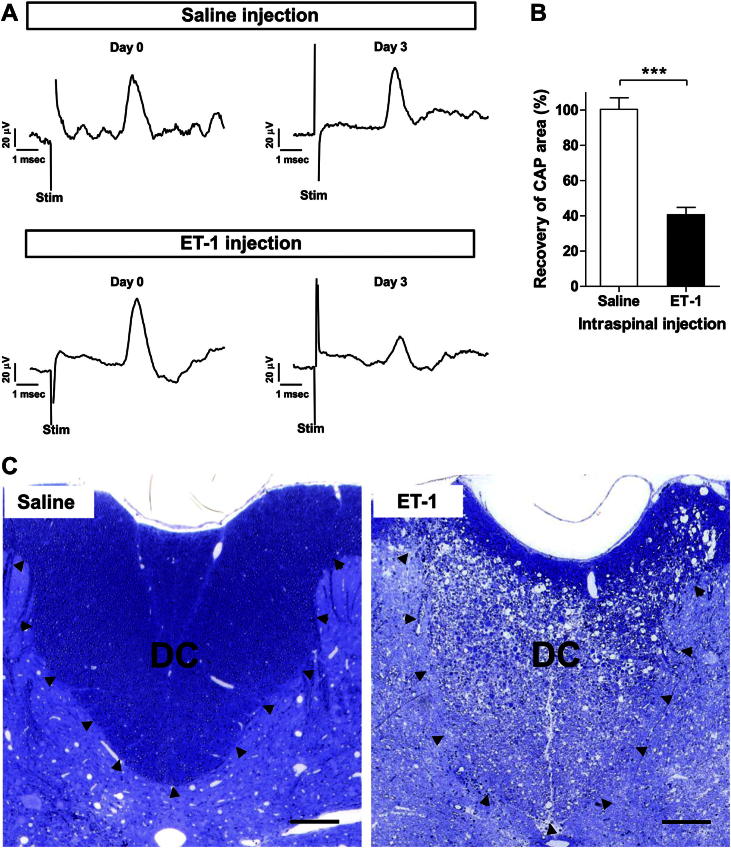
Data showing the recovery model of ET-1-induced ischemia. The initial ischemia was induced by bilateral injections of 2.28 nmol ET-1 into the gray matter, followed by a second set of injections of 1.14 nmol administered 60 min after the initial set. A shows representative, averaged compound action potentials (CAPs) evoked by supramaximal stimulation in the same animals at day 0 (pre-injection) and at day 3 post-injection, with saline or ET-1 treatment. B shows the averaged percentage of the area under the post-injection CAP relative to that under the pre-injection CAP, following ET-1 (40.6 ± 11%, *n* = 6) or saline injection (100.4 ± 7%, *n* = 5). ET-1 significantly reduced the area of the CAP conducted along the dorsal column axons. C shows transverse sections of the dorsal columns (DC; outlined by arrowheads), 3 days after either saline or ET-1 injection. Whereas the tissue appears normal following saline injection, there is substantial degeneration of the dorsal column axons following ET-1 injection, except for a thin band beneath the pial surface. Stim: stimulus artifact. Data in B: mean ± SD. ***P < 0.001, Student's *t* test. Bars in *C* = 200 μm.

Indeed, examination of animals three days after intraspinal injection revealed that ET-1-induced axonal injury persisted, as the area under the CAP remained significantly reduced (40.6 ± 11% of that before ET-1 injection, *n* = 6, Fig. 2A & B), whereas similar saline control injection had little or no long term effect (100.4 ± 7% of that before saline injection, *n* = 5, Fig. 2A & B). Histologically, the three day period following the ischemic insult allowed the degenerating axons to be easily distinguished such that there was a clear difference between the infarcted and surviving regions of tissue (Fig. 2C). The lower portion of the dorsal columns was most severely affected, and in all cases the most superficial tissue was relatively preserved. At the ‘border’ between these regions there was a mixture of seemingly normal axons, and axons that were undergoing demyelination or degeneration. Saline injection had very little effect on the integrity of the dorsal columns (Fig. 2C).

### 3.3 Protection of axonal function: acute preparation

**Fig. 3:**
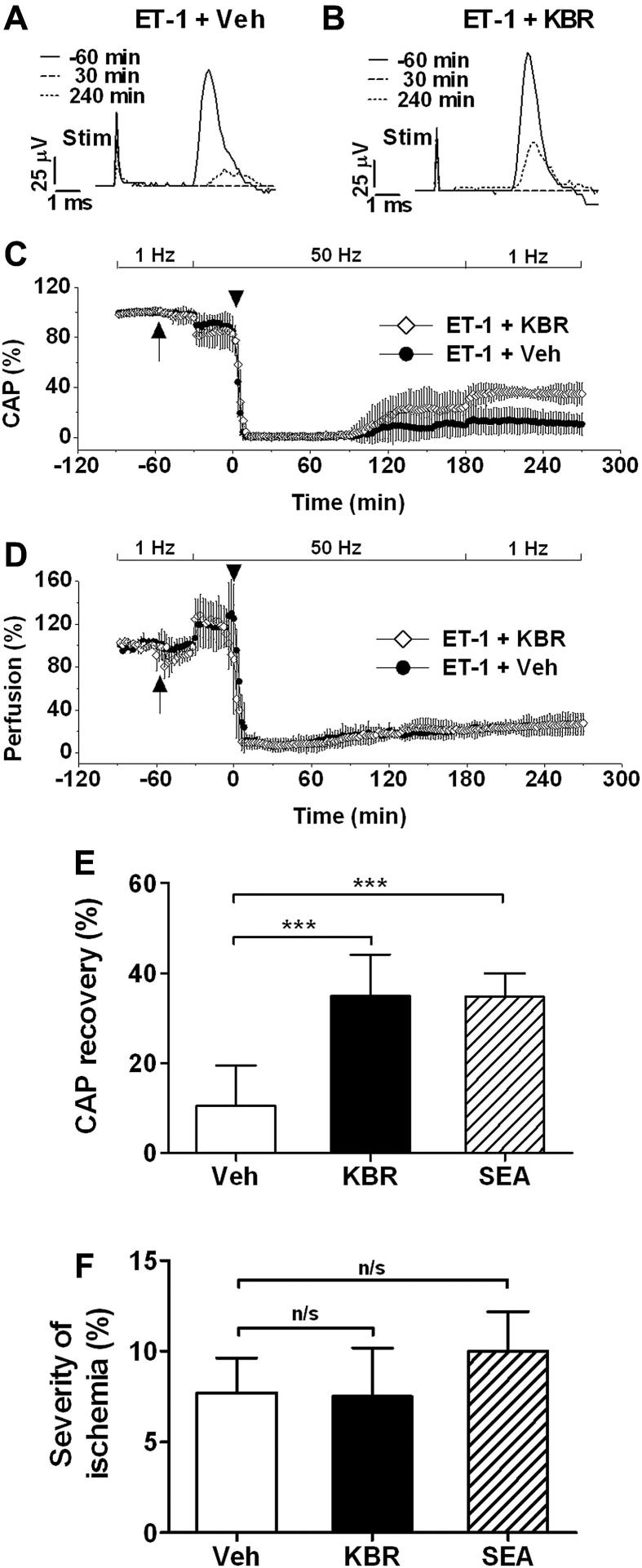
Data showing the protection of axonal function by KB-R7943 and SEA0400 in the acute experiment. A & B show representative CAPs obtained from animals injected with ET-1 and treated with either vehicle (A) or KB-R7943 (B). The response attained at the start of recording (solid line) indicates the maximal number of axons conducting in response to the supramaximal electrical stimulus, but 30 min following ET-1 injection no conducting axons could be detected (dashed line). In animals treated with KB-R7943, the CAP obtained four hours after ET-1 injection was noticeably greater in amplitude and area than that obtained from saline-treated controls (dotted line). Stim: stimulus artifact. C shows similar findings, plotted against time, from all the animals studied. The systemic administration of KB-R7943 or saline (arrow) had no immediate detectable effect on the CAP, but there was a small reduction in the amplitude of the CAP upon the onset of stimulation at 50 Hz, and a prompt reduction to zero upon the injection of ET-1 (arrowhead). Conduction resumed at approximately the same time following ET-1 (∼100 min) irrespective of therapy, but in the animals treated with KB-R7943 the recovery was significantly greater, as represented graphically in E. D shows changes in the relative perfusion of the spinal cord at the level of the lesion as monitored continuously using laser Doppler flowmetry. The administration (arrow) of either KB-R7943 (KBR; 30 mg/kg, i.a.) or vehicle (Veh, i.a.) was associated with a brief and mild reduction in perfusion, and the onset of 50 Hz stimulation caused a more pronounced increase in perfusion. Perfusion was promptly reduced to less than 10% upon the intraspinal injection of ET-1 (2.28 nmol) (*t* = 0). E illustrates the significant improvement in recovery of axonal function achieved by treatment with KB-R7943 or SEA0400, as judged by the amplitude of the conducted CAP (Veh 10.5 ± 9% of baseline, *n* = 8; KBR 35.0 ± 9% of baseline, *n* = 6, *P* < 0.001; SEA 33.2 ± 6% of baseline, *n* = 4, *P* < 0.001). F shows the minimal level of perfusion achieved with Veh (7.7 ± 4% of baseline, *n* = 5), KBR (7.5 ± 5% of baseline, *n* = 4) or SEA (10.0 ± 4% of baseline, *n* = 4). The severity of ischemia induced by ET-1 was not reduced by the drug treatments. Data: mean ± SD. ***P < 0.001, compared with vehicle, one-way ANOVA with Bonferroni *post hoc* test.

The administration of KB-R7943 (30 mg/kg, i.a.) sometimes appeared transiently to reduce perfusion of the spinal cord (Fig. 3D), but the drug had no discernable effect on the CAP prior to the onset of ischemia, nor did the drug significantly affect the timing or magnitude of the loss of conduction upon ET-1 administration. However, notably, the drug significantly improved the recovery of the CAP following ischemia, in comparison with vehicle treatment (35.0 ± 9% of baseline for KB-R7943, *n* = 6, vs. 10.5 ± 9% for vehicle, *n* = 8; *P* < 0.001; Fig. 3A, B, C & E). Importantly, the drug had no significant effect on the severity of ischemia following ET-1 (perfusion reduced to 7.5 ± 5% of baseline for KB-R7943 vs. 7.7 ± 4% of baseline for vehicle; *P* > 0.05; Fig. 3D & F), showing that the protective effect of KB-R7943 on axonal conduction was not due to a diminution of the severity of ischemia.

In common with KB-R7943, treatment with SEA0400 (30 mg/kg, i.a.) also resulted in an increased recovery of the CAP (33.2 ± 6% of baseline, *n* = 4) compared with vehicle treatment (10.5 ± 9% of baseline, *n* = 8; *P* < 0.001; Fig. 3E). Again, the protection was achieved without diminishing the severity of ischemia (Fig. 3F).

### 3.4 Protection of axonal function: recovery preparation

**Fig. 4:**
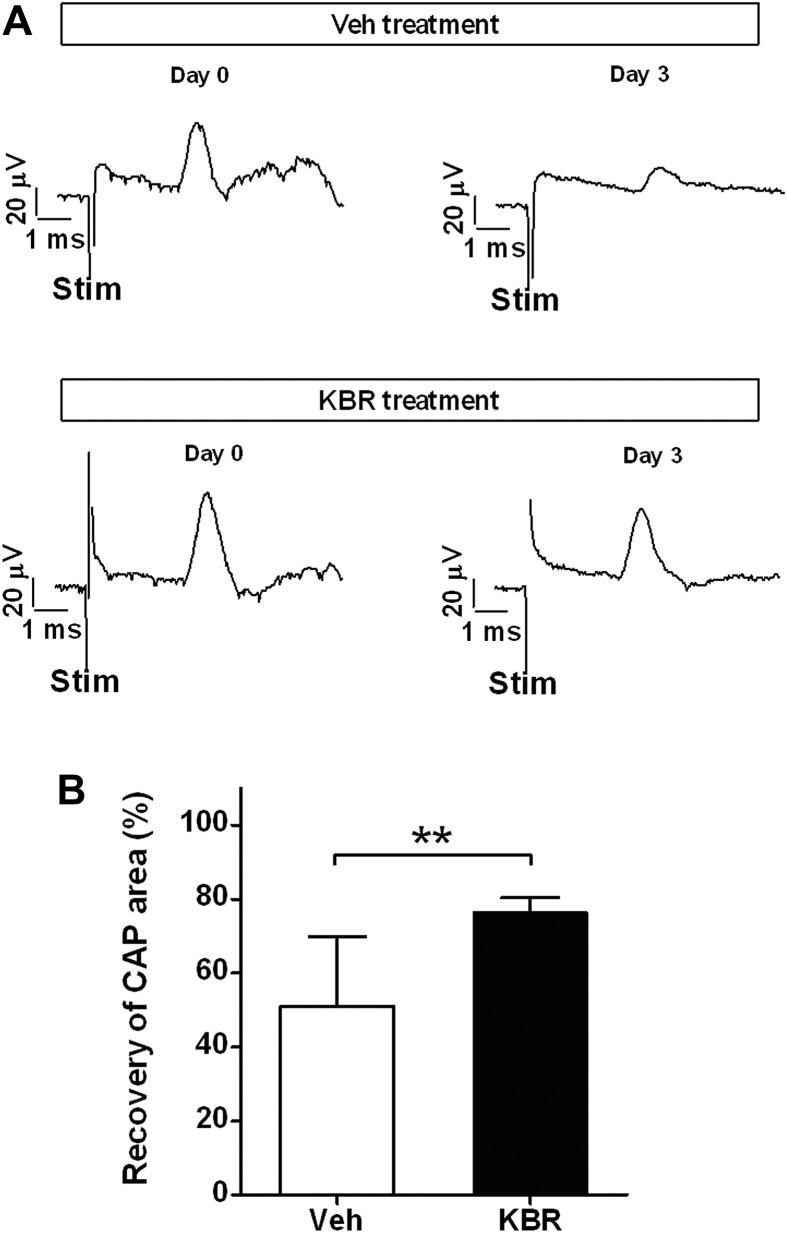
Data showing the protection of axonal function by KB-R7943 in the recovery experiment. A shows representative, averaged (*n* = 32) CAPs recorded from the same animals before the induction of ischemia on day 0, and three days later, in response to supramaximal electrical stimulation. Therapy with KB-R7943 (KBR, 30 mg/kg, i.v.) administered 60 min prior to the initial ET-1 injection resulted in an increase in the area under the CAP compared with vehicle (Veh, 40% DMSO in saline) when measured after three days. B illustrates the area under the CAP at day 3 post-injection, expressed as a percentage of the area at day 0 pre-injection, with KBR treatment (76.3 ± 11%, *n* = 8) or Veh treatment (51.0 ± 19%, *n* = 8). Stim: stimulus artifact. Data: mean ± SD. **P < 0.01, Student's *t* test.

To determine whether the protection of axonal function observed in the acute experiments represented a meaningful, i.e. sustained, protection of function and structural integrity (rather than, perhaps, a delay in degeneration), other experiments applied electrophysiological and histological examinations to assess the axons after a delay of three days following the ischemia. In these experiments the severity of the lesion was increased by a second intraspinal injection of ET-1 (equal to half the first dose), rather than the use of high frequency electrical stimulation. Treatment with KB-R7943 (30 mg/kg, i.v.) significantly increased the area under the CAP (76.3 ± 11% of baseline, *n* = 8) at day 3 after ET-1 injection, compared with vehicle treatment (51.0 ± 19% of baseline, *n* = 8; *P* < 0.01), as shown in Fig. 4.

**Fig. 5:**
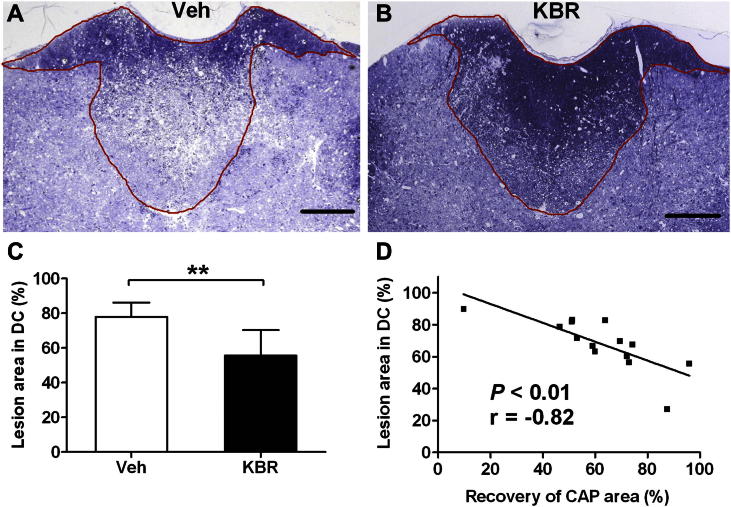
Protection of axonal integrity by KB-R7943 in the recovery experiment. A and B show representative sections of the dorsal columns (outlined in solid line) at the site of injection of ET-1 in animals treated with vehicle (Veh) or KB-R7943 (KBR), 3 days after injection. Regions with surviving axons appear dark (at this magnification), and degenerating tissue appears pale. Sections were treated with osmium tetroxide and stained with 1% toluidine blue. C shows the area of the lesion expressed as a percentage of the area of the whole dorsal columns: animals treated with KBR had significantly smaller lesions in the dorsal columns (55.6 ± 15%, *n* = 6) than Veh-treated controls (77.9 ± 9%, *n* = 8, *P* < 0.01). D: graph of the area of the lesion in the dorsal columns, expressed as a percentage of the area of the whole dorsal columns, vs. the area under the CAP, expressed as a percentage of the area obtained prior to ET-1 injection (Spearman's rank correlation test, *P* < 0.01, *r* = −0.820). DC: dorsal columns. Bars in A&B = 300 μm. Data in C: mean ± SD. **P < 0.01, Student's *t* test. (For interpretation of the references to color in this figure legend, the reader is referred to the web version of this article.)

In agreement with the electrophysiological findings, treatment with KB-R7943 significantly reduced the area of pathology in the dorsal columns (55.6 ± 15%, *n* = 6) compared with vehicle treatment (77.9 ± 9%, *n* = 8; *P* < 0.01; Fig. 5A–C). There was a significant negative correlation between the lesion area and the area under the CAP (*P* < 0.01, *r* = −0.820, Spearman's rank correlation test; Fig. 5D).

## 4 Discussion

A novel *in vivo* model of transient focal ischemia has been introduced and used to explore whether inhibition of the reverse-mode operation of the NCX is effective in protecting the white matter from ischemic degeneration. Both of the NCX inhibitors KB-R7943 and SEA0400 were found significantly to improve the number of functional axons in the dorsal columns following ischemia, as assessed by the magnitude of the conducted CAP (increased by ∼200% above control in the acute experiments, and by ∼50% in the recovery experiments). The axonal protection was evident soon after the period of ischemia, and it persisted for at least 3 days. Correspondingly, histological examination revealed that the area of axonal degeneration in the dorsal columns was also significantly reduced (by 29% 3 days after intraspinal injection) in the treated animals.

### 4.1 Ischemia model

The new model comprises a lesion involving both the gray and white matter, but the model may be especially valuable for studying the white matter. For example the function of the affected axons in the dorsal columns can be monitored electrophysiologically, serially over days, to assess the time course of axonal damage and recovery, and the efficacy of strategies for axonal protection. This ability to assess axonal function serially is of particular interest, since no such assessment of axonal function after ischemia has been reported *in vivo* to our knowledge. The antidromic conduction pathway described here, comprising the dorsal columns, spinal roots, and peripheral nerves includes the site of the dorsal column lesion (of course), but the cell bodies of the affected axons are located remotely from the ET-1 injection site (within the dorsal root ganglia) and so may be expected to be spared from direct ischemic damage. This feature allows the effects of the ischemia on the axons to be studied in the absence of confounding effects arising from direct damage to the associated cell bodies. The protocol to induce the lesion involves only minimal mechanical damage to the spinal tissue, such that control animals show no electrophysiological deficits and only a few degenerating axons (sometimes apparent along the injection track upon microscopic examination in high resolution resin sections). In contrast to other, related studies (e.g. Frost et al., 2006; Sozmen et al., 2009), the protocol adopted here avoids the injection of ET-1 directly into the white matter, thereby reducing the possibility of any ‘direct’ effect of ET-1 on glial cells or axons. However, we have in fact found no direct effect of ET-1 on axonal conduction upon direct injection of concentrated ET-1 into the dorsal columns themselves. Indeed, we found that while ET-1 injection into the dorsal columns was inefficient in inducing persisting acute axonal injury, ET-1 injection into the gray matter adjacent to the dorsal columns resulted in a profound reduction of blood perfusion within the dorsal columns, causing ischemia and the profound electrophysiological and structural damage.

A useful feature of the method is that the severity of the lesion can be modulated in two ways, either by changing the frequency or duration of electrical stimulation, or by repeating the injection of ET-1 as vascular perfusion starts to return. The first method is especially useful as the insult is both convenient to administer, and very easily graded.

The acute and recovery preparations are two integrated parts of the model. The acute preparation allows for continuous monitoring of the integrity of axonal function during the initial phase of ischemia, including monitoring of physiological parameters such as local vascular perfusion. This facility permits distinction between whether a axonal protection is achieved by “direct” mechanisms (i.e. by acting on axons) or by “indirect” mechanisms (e.g. by alleviating the ischemia) or both. The recovery preparation, clinically more relevant, can be used to validate the acute findings by determining whether any axonal protection is durable over days.

Histological examination of the lesioned tissue suggested the presence of a gradient of vulnerability within the dorsal columns along the dorso-ventral axis, with the ventral portion being most severely affected. It seems likely that the more dorsal tissue received some vascular-derived sustenance from the pial plexus (Tveten, 1976), and even possibly from the cerebrospinal fluid and dorsal vein. If so, the new lesion may be helpful in modeling the penumbra observed clinically in the white matter (Koga et al., 2005; Murphy et al., 2008), and the efficacy of different drugs in neuroprotection can be revealed by how far ventrally in the dorsal columns the border between surviving and degenerating tissue can be extended.

### 4.2 Axonal protection

The NCX is a bi-directional transporter that plays an important role in the regulation of the axoplasmic concentration of Ca^2+^ and Na^+^ (Baker and McNaughton, 1976; Blaustein and Santiago, 1977). The strategy of blocking the reverse-mode action of the NCX to achieve axonal protection from ischemia was introduced in studies by Stys, Waxman, Ransom and colleagues who removed oxygen, or oxygen and glucose, from excised tissue studied *in vitro* (Stys et al., 1992b; Ouardouz et al., 2005). The present model has permitted examination of whether the therapeutic strategy is both feasible and effective in a true ischemic lesion studied *in vivo*. The current findings show that both KB-R7943 and SEA0400, selective inhibitors of the reverse-mode action of the NCX, provide significant protection of axons from ischemic degeneration *in vivo*.

KB-R7943 has a broad pharmacological profile (See review Annunziato et al., 2004). Apart from inhibiting the NCX, it is also reported to inhibit L-type calcium channels (Ouardouz et al., 2005), NMDA receptors (Sobolevsky and Khodorov, 1999), the Na^+^/Mg^2+^ exchanger (Uetani et al., 2003), and nicotinic acetylcholine receptors (Pintado et al., 2000). Indeed, the inhibition of the L-type Ca^2+^ channel by KB-R7943 may contribute to the axonal protection observed in this study as pharmacological inhibition of the L-type Ca^2+^ channel has been shown to protect the optic nerve axons from anoxic injury (Fern et al., 1995) by reducing the Ca^2+^ influx through the channel and/or by reducing the Ca^2+^ release from the internal store (Ouardouz et al., 2003). However, the fact that the protective effect of KB-R7943 is reproduced by another more specific inhibitor of the reverse-mode action of the NCX, SEA0400 (Matsuda et al., 2001), strengthens a belief that this shared property of the drugs is important in their neuroprotective mode of action.

Pre-treatment with KB-R7943 provided robust protection of the dorsal column axons, as assessed by both electrophysiological and histological methods. A potential concern is that the protection was misleadingly achieved by diminishing the severity of the ischemic insult, but this possibility was discounted by the observation from the laser Doppler flowmetry that blood perfusion was not improved by the drug, and in fact rather transiently reduced. Another potential concern is that KB-R7943 might misleadingly increase the amplitude of the CAP by some unsuspected mechanism, giving the misleading impression of an increase in the number of conducting axons, but this possibility was also discounted by the observation that the same dose of KB-R7943 did not change the waveform of the CAP in naïve animals (data not shown). It is also theoretically possible that KB-R7943 achieved protection by inducing hypothermia (Pignataro et al., 2004), as this state can be neuroprotective (Stys et al., 1992a). However, in the acute experiments the core temperature of the rats was maintained at a stable level (36.5 ± 0.5 °C) throughout the experiment by external heat, so hypothermia is unlikely to explain the axonal protection observed in this study. We conclude that the findings obtained with KB-R7943 administration are indeed due to protection of the axons from the consequences of the ischemic insult.

In summary, a new model of white matter ischemia has been developed in the adult rat spinal cord using vasoconstriction induced by the intraspinal injection of ET-1. The model has been used to demonstrate that two drugs that inhibit the reverse-mode action of the NCX, KB-R7943 and SEA0400, are effective in protecting axons from ischemic injury *in vivo*, and that the protection persists for at least 3 days.
