# Supplementary material for: Axonal protection achieved by blockade of sodium/calcium exchange in a new model of ischemia in vivo
Source: Neuropharmacology. 2012 Sep;63(3):405–14. doi: 10.1016/j.neuropharm.2012.04.019 (PMC3657694; doi:10.1016/j.neuropharm.2012.04.019)
Supplement: Supplementary file 1 [file mmc1.docx]

A

**1 Hz**

**F**

**1 Hz**

**-90 min**

**-30 min**

**180 min**

**270 min**

**Time (T)**

**Stimulation frequency**

B

Supplementary Fig. 1. Electrical activity and axonal injuries induced by ET-1. To examine the effects of both ET-1 injection and electrical activity on axonal conduction, intraspinal injection of ET-1 (2.28 nmol; T = 0) was performed in the spinal gray matter adjacent to the dorsal columns, and axons were either physiologically challenged with 50 Hz electrical stimulation or not (i.e. 1 Hz baseline stimulation only) (A). Both the stimulating frequency and the injection significantly affected the recovery of the CAP (*P* <0.001). ET-1 injection caused significantly less recovery of the CAP for axons after 50 Hz stimulation (18.5 ± 11%, n = 5) in comparison with axons after 1 Hz stimulation alone (56.2 ± 8%, n = 3, *P* <0.001). Saline injection resulted in 97.2 ± 2% (n = 3) and 92.8 ± 4% (n = 3) of recovery after 1 and 50 Hz stimulation respectively. Data: mean ± SD. ****P* < 0.001, two-way ANOVA with Bonferroni *post hoc* test.

**50 Hz**

**1 Hz**

**1 Hz**

Supplementary Fig. 2. Intraspinal injection of ET-1 temporarily affects the systemic blood pressure in anaesthetized rats. The blood pressure (mean arterial pressure) of each rat was recorded every 10 minutes along with the continuous monitoring of axonal conduction in the dorsal columns. ET-1 injection (2.28 nmol, n = 5; arrowhead) at the spinal gray matter (GM) reduced the blood pressure by 0-50% (23 ± 24%), but this always returned to baseline within 150 minutes of ET-1 injection. No significant difference of the blood pressure was observed between the ET-1 and saline groups (n =3) at any time point recorded (Student’s *t* test). Data: mean ± SD.


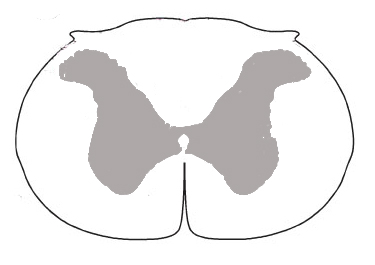


**ET-1**

**DC**

**1 Hz**

**50 Hz**

**1 Hz**

Supplementary Fig. 3. Examination on the “neurotoxicity” of ET-1 by directly exposing dorsal column (DC) axons to ET-1. ET-1 of at the maximal concentration (0.28 mM) was directly injected into the DC. The injection caused a reduction in the amplitude of the CAP after a longer delay than that typically observed when ET-1 was injected into the gray matter. Axonal conduction in the DC was transiently blocked by the DC injection, with final recovery of the amplitude to 78.8 ± 15% of baseline (n = 3). The transient blockade of axonal conduction was likely due to ischemia resulting from the diffusion of ET-1 into the adjacent gray matter, which provides a sufficient explanation for the incomplete recovery of conduction. These findings provide no evidence to support a major “neurotoxicity” of ET-1 in causing acute conduction blockade. Data: mean ± SD. Arrowhead: intraspinal injection at t = 0.

Supplementary Fig. 4. Estimated change of local blood perfusion after ET-1 injection in recovery experiments. As measured by laser Doppler flowmetry, the local blood perfusion was reduced by ET-1 injection (n = 6), and recovered to ~80% by 1 day after injection. No significant improvement of blood perfusion was observed between day 1 and day 3. Intraspinal injection of saline also transiently reduced the blood perfusion, although to a lesser extent compared with ET-1 injection. Arrowhead: onset of intraspinal injection at t = 0.
